# Event-related EEG power modulations and phase connectivity indicate the focus of attention in an auditory own name paradigm

**DOI:** 10.1007/s00415-016-8150-z

**Published:** 2016-05-23

**Authors:** Julia Lechinger, Tomasz Wielek, Christine Blume, Gerald Pichler, Gabriele Michitsch, Johann Donis, Walter Gruber, Manuel Schabus

**Affiliations:** 1 Laboratory for Sleep and Consciousness Research, Department of Psychology, University of Salzburg, Hellbrunnerstraße 34, 5020 Salzburg, Austria; 2 Centre for Cognitive Neuroscience (CCNS), University of Salzburg, Salzburg, Austria; 3 Apallic Care Unit, Neurological Division, Albert-Schweitzer-Klinik, Graz, Austria; 4 Apallic Care Unit, Neurological Division, Sozialmedizinisches Zentrum Ost-Donauspital, Vienna, Austria

**Keywords:** Disorders of consciousness, Auditory own name paradigm, EEG, Time–frequency analysis, Phase connectivity

## Abstract

Estimating cognitive abilities in patients suffering from Disorders of Consciousness remains challenging. One cognitive task to address this issue is the so-called own name paradigm, in which subjects are presented with first names including the own name. In the active condition, a specific target name has to be silently counted. We recorded EEG during this task in 24 healthy controls, 8 patients suffering from Unresponsive Wakefulness Syndrome (UWS) and 7 minimally conscious (MCS) patients. EEG was analysed with respect to amplitude as well as phase modulations and connectivity. Results showed that general reactivity in the delta, theta and alpha frequency (event-related de-synchronisation, ERS/ERD, and phase locking between trials and electrodes) toward auditory stimulation was higher in controls than in patients. In controls, delta ERS and lower alpha ERD indexed the focus of attention in both conditions, late theta ERS only in the active condition. Additionally, phase locking between trials and delta phase connectivity was highest for own names in the passive and targets in the active condition. In patients, clear stimulus-specific differences could not be detected. However, MCS patients could reliably be differentiated from UWS patients based on their general event-related delta and theta increase independent of the type of stimulus. In conclusion, the EEG signature of the active own name paradigm revealed instruction-following in healthy participants. On the other hand, DOC patients did not show clear stimulus-specific processing. General reactivity toward any auditory input, however, allowed for a reliable differentiation between MCS and UWS patients.

**Electronic supplementary material:**

The online version of this article (doi:10.1007/s00415-016-8150-z) contains supplementary material, which is available to authorized users.

## Introduction

After severe brain injury, some patients do not or not fully regain consciousness. The former are considered unresponsive, i.e., suffer from an Unresponsive Wakefulness Syndrome (UWS), meaning they open their eyes spontaneously but do not show any sign of consciousness of themselves or their environment. The latter group is diagnosed with Minimally Conscious State (MCS), which actually subsumes a broad range of patients who additionally show weak behavioural signs of consciousness such as visual pursuit or the ability to follow simple commands. Differentiating these two so-called “Disorders of Consciousness” (DOC) diagnoses and evaluating and appraising the extent to which some patients are still able to experience their environment remains challenging. The clinical diagnosis is usually based on the evaluation of the patients’ behavioural presentation in response to stimulation. This approach, however, has repeatedly proven to be prone to a high rate of misdiagnoses [1, 8, 40], which can affect patient care and raises ethical and legal questions. The scientific approach to studying DOC has, therefore, tried to address this problem by including neuroscientific, i.e., neuroimaging and neurophysiological methods. The underlying rationale is that such approaches may reveal brain responses to certain types of stimuli that are not necessarily captured in behavioural assessments.

A popular task that has been introduced to this field of research several years ago is the so-called “own name paradigm” in which participants are presented with an auditory sequence of first names, including the subject’s own name (SON; [35, 39]). A person’s own first name is an extremely salient stimulus that immediately draws the person’s attention, which was even shown to result in a P3 response in DOC patients [35, 39]. Beyond ERPs, the SON has also been shown to lead to stronger even-related desynchronization (ERD) in the EEG alpha band in control subjects as compared to the SON spoken backwards or an unfamiliar name [17]. In UWS and MCS patients stronger activation in higher auditory areas of the temporal cortex has been observed for the SON [10]. Interestingly, UWS patients with the most widespread activity progressed to an MCS within the following months. Chen et al. [7] conducted a similar task in healthy participants and presented terms with a graded extent of self-reference (e.g., own name as highly self-relevant, name of their province as moderate self-relevant). They were able to show that the extent of self-reference was positively associated with P3 amplitude and peak latency, and negatively correlated with the N2 amplitude.

However, due to the own name’s special relevance to oneself and its bottom–up strength, its presentation elicits responses even in (presumably) unconscious states such as sleep. Perrin et al. [34] found enhanced early components of K-complexes (especially the positive component at about 600 ms) in non-rapid eye movement sleep stage 2 (NREM 2) and rapid eye movement sleep (REM) as well as an increase in alpha band power during NREM 2 for the own name compared to the other names. Based on these findings, brain activation in response to a passive presentation of the SON seems to entail rather sparse information about the extent of cognitive abilities in DOC patients, let alone consciousness.

To overcome these limitations, a higher-order cognitive component has been added to refine the paradigm. In an active version of the own name task, DOC patients were not only asked to listen to a sequence of names, but also count the number of appearances of their own and another unfamiliar name [39]. It has been shown that in both controls and MCS patients the event-related P3 response was more pronounced for the names that had to be counted as compared to those that had only been listened to. A follow-up analysis revealed that all patients showed a higher event-related synchronisation (ERS) in the theta frequency band (4–7 Hz) above frontal regions when they were supposed to actively count the SON as compared to when they were only listening to it [12]. This is coherent with the assumption that frontal activation in the theta band is an indicator of top–down attentional modulation, which is necessary for an active demand such as counting. Likewise, in control subjects alpha ERD was also more pronounced when names were to be counted than when participants simply listened to them.

Beyond local activations in specific regions, control subjects and MCS patients—compared to UWS patients—also showed stronger connectivity [measured by positron emission tomography (PET)] between auditory and frontal association cortices while being presented with the SON [3]. On the other hand, Monti et al. [31] tested one MCS patient and found simultaneous activation of temporal, parietal and frontal regions when the patient was supposed to count an unfamiliar name, but not when she just passively listened to it. Thus, an activation of frontal cortices either seems to occur when modulation by attention is needed, or when the stimulus is as salient as the SON and, therefore, possibly related to automatic attention orientation and possibly self-referential processing [11, 13].

In the present study, we recorded EEG during an own name task in a group of UWS and MCS patients as well as healthy controls and concentrated on a more fine-grained analysis of the EEG signal which also focused on the timing in information processing as well as connectivity with respect to different frequency bands. Specifically we analysed event-related EEG power changes, together with inter-trial and inter-electrode phase coherence in the delta (0.5–3 Hz), theta (3.5–6.5 Hz) and lower alpha (7.5–9.5 Hz) frequency band. While inter-trial coherence focuses on the exact timing of brain processing from trial to trial, inter-electrode phase coherence is a connectivity measure addressing the stable timing between scalp sites across trials. By including time-locked and non-time-locked responses to auditory stimulation, we aimed at having a more sensitive analysis in a group of severely impaired patients, in whom the exact timing of brain responses similar to healthy cannot necessarily be expected.

## Methods and materials

### Subjects

Two groups of patients, one MCS group (*N* = 7, mean age = 47.43 years, SD = 16.19 years) and one UWS group (*N* = 8, mean age = 48.13 years, SD = 11.24 years, please refer to Table 1), were tested. The aetiology of brain damage was either traumatic (cerebral haemorrhage or direct trauma to the head) or non-traumatic (cerebral hypoxia or, in one patient, by subacute sclerosing panencephalitis). Diagnosis was established by two independent raters using the Coma Recovery Scale—Revised [22]. All patients were in a stable and persistent state that lasted a minimum of eight months (mean = 71.00 months, SD = 51.00 months). For every patient informed consent was obtained from relatives or legal representatives. Our control group consisted of 24 healthy age and sex matched volunteers (*N*_male_ = 9, *N*_female_ = 15, mean age = 46.04 years, SD = 14.52 years). All participants were native German speakers. Ethical approval was obtained from the ethics committee of the Medical University of Graz. The study was performed in accordance with the Declaration of Helsinki and Good Clinical Practice Guidelines.

**Table 1:** Demographic data of patients with Minimally Conscious State (MCS) and Unresponsive Wakefulness Syndrome/Vegetative State (UWS/VS)

Patient ID	Age (years)	Sex	Aetiology	Time since injury	Clinical assessment	CRS-R total score	CRS-R auditory score
MCS1	57	m	Anoxic brain lesion after myocardial infarction	11 years 3 months	MCS	12	3
MCS2	45	m	Subdural hematoma, subarachnoidal haemorrhage, skull fracture	1 year	MCS	8	0
MCS3	56	w	Hypoxia	7 years 1 month	MCS	12	3
MCS4	73	m	Intracerebral haemorrhage	8 months	MCS	17	3
MCS5	21	m	Anoxic brain lesion after mixed intoxication	2 years 4 months	MCS	13	3
MCS6	50	w	Subdural hematoma after violent crime	9 years 5 months	MCS	14	4
MCS7	30	m	Trauma	9 years 3 months	MCS	13	3
UWS1	20	m	SSPE (syn. Bogaert encephalitis)	3 years	UWS	3	1
UWS2	51	w	Subdural hematoma, ruptured aneurism, hydrocephalus	4 years 1 month	UWS	4	0
UWS3	48	m	Hypoxia	9 years 3 months	UWS	5	0
UWS4	52	m	Subdural hematoma, osteoclastic trepanation	12 years 3 months	UWS	8	2
UWS5	53	m	Trauma	1 year 1 month	UWS	4	0
UWS6	58	w	Ruptured aneurism	2 years 4 months	UWS	4	0
UWS7	62	m	Hypoxia after cardiopulmonary resuscitation	2 years 8 months	UWS	4	2
UWS8	41	w	Deceleration trauma with cortical and subcortical contusions	12 years 8 months	UWS	7	2

Please note that this data set was also recently analysed using entropy measures [45].

### Experimental procedure

The task comprised two conditions in which patients and healthy controls were binaurally (over headphones) presented with five different first names. One name was the SON, the others were names that are very common in Austria, but supposedly not emotionally relevant to the subject (i.e, no names of relatives, close friends or primary nursing staff were used). In the first (passive) condition subjects were instructed to only listen to the presented names. In the second (active) condition subjects were told to concentrate on a specified other name and silently count the number of its occurrences. Every recording session started with the passive listening condition. The active condition’s target name was always also included in the previous passive condition’s stimulus set. Other names were matched with the SON regarding syllable count within each patient. Every name was presented 45 times per condition in a randomised order. The inter-stimulus interval (ISI) was 3 s. Patients completed the task twice on two different occasions separated by an interval of 2–4 weeks.

### EEG acquisition and stimulus presentation

EEG was recorded from 19 scalp positions using Ag/AgCl electrodes with a BrainAmp (Brain Products, Gilching, Germany) amplifier at 1000 Hz sampling rate. Impedances were kept below 5 kΩ. Scalp positions were F3, F4, FC5, FC6, C3, C4, P3, P4, T3, T4, F7, F8, PO7, PO8, Fz, Cz, Pz, Oz and FCz according to the international 10–20 system [20]. Online, the signal was referenced against FCz, and later re-referenced against averaged mastoids. Vertical and horizontal electrooculogram (EOG) was recorded using four electrodes. For electromyography (EMG) two electrodes were placed on and below the chin, respectively. Furthermore, electrocardiography (ECG) and respiration were recorded.

### EEG analysis

Following re-referencing, the EEG signal was downsampled to 500 Hz and bandpass-filtered between 1 and 40 Hz using an infinite impulse response (IIR) filter with a slope of 48 dB. Ocular corrections were conducted using Gratton and Coles correction as implemented in Brain Vision Analyzer 2.0 (Brain Products, Gilching, Germany). The signal was checked visually for myogenetic and other remaining artefacts. For ERS/ERD and inter-trial phase locking (phase-locking index, PLI, as described in Schack et al. [38], and Tallon-Baudry et al. [44]) analysis the continuous EEG signal was filtered using a phase-shift-free Butterworth filter (slope 24 dB/oct) in three frequency bands [delta (1–3), theta (3.5–7 Hz), lower alpha (8–10 Hz)] and segmented into 1.2 s epochs ranging from −400 to +800 ms with respect to stimulus onset. We restricted our analyses to the delta–alpha frequency range, because already in healthy controls beta frequency and above are often contaminated by muscle artefacts [14] and even more so in DOC patients who often present with spasticity (e.g., high muscle tone). Former studies from our group indicated that the delta to alpha range presents with the most reliable results, while beta is contaminated by myogenic artefacts [27]. Last, the EEG spectrum of DOC patients is often shifted toward slow frequencies [24, 25].

PLI was calculated over trials and ERS/ERD [36] was calculated after averaging rectified segments. The interval from −200 to 0 ms relative to stimulus onset was chosen as baseline.

For inter-electrode phase locking, the phase-locking value (PLV, [26]) was calculated. For PLV analysis the continuous signal was current source density transformed (order of splines: 4, maximal degree of Legendre polynomials: 20, approximation parameter Lambda: 1e−5) prior to filtering to account for volume conduction. The PLV was calculated for all 171 possible electrode pairs resulting from 19 electrodes.

PLI and PLV take into account oscillatory phase only and are mostly insensitive to power modulations. Both measures range from 0 to 1, with 0 reflecting no coherence and 1 indicating maximal phase synchronicity between trials (PLI) or between scalp sites (PLV). While inter-trial coherence focuses on the exact timing from trial to trial, inter-electrode phase coherence is a connectivity measure addressing the stable timing between brain areas across trials without time lag. Please note that the PLI (especially in the higher the frequencies) might be sensitive to the physical properties of the stimuli. We tried to account for this possibility by treating the name which was to become the target in the active condition as a separate stimulus in the analysis of the passive condition already. We only considered a target vs. other name difference a real effect, when it was present in the active condition, but not evident as a “target-to-be vs. other name effect” in the passive condition.

### Statistical analysis

#### ERD/ERS and PLI

In a first step we were interested whether the different stimuli elicit different delta, theta and alpha ERS (ERD) and PLI within the control group. ERS and PLI values were subjected to ANOVAs with the factors STIMULUS [own name vs. target (subsequent) vs. other names] × TIME (0–200 vs. 200–400 vs. 400–600 vs. 600–800 ms) for the passive and the active condition. In the passive conditions we decided to include the one unfamiliar name, which will subsequently become the target in the active condition, as a separate stimulus into the analysis in order to have the same statistical power between passive and active condition. For post hoc comparisons, *t* tests were calculated and corrected for multiple comparisons according to Benjamini and Hochberg [2].

In a next step, we additionally tested for group differences extending the ANOVAs by a between factor GROUP (control vs. MCS vs. UWS). *p* values below .05 were considered significant and *p* values below .10 will be mentioned as tendencies.

Furthermore, for PLI analysis, the distribution of phase values was evaluated using Rayleigh tests (null hypothesis: uniform distribution; critical *α* < .05) in order to test if the resulting PLI was meaningful or spurious. Results of Rayleigh tests will be reported to substantiate relevant ANOVA/*t* test results.

#### PLV

To test for significant changes in test intervals relative to baseline a bootstrapping procedure based on paired samples *t* tests was conducted. As baseline, the interval from −200 to 0 ms was used. As test intervals, four successive 200 ms time windows after stimulus onset were averaged in order for the analysis to be comparable to the ERS/PLI analysis. The bootstrapping procedure, which was applied to each time window for the three entities (controls, MCS, UWS), was the following: The real data were permuted 10,000 times per group (control, MCS and UWS). From the resulting distribution of *p* values for each electrode pair the 5th percentile was defined as the criterion. Subsequently, *t* tests were calculated again on the real data and the resulting *p* values were compared to the critical value. Only those differences between test and baseline interval that presented a *p* value below the critical *p* for that electrode pair were considered significant. Analyses were again conducted for the three frequency bands (i.e., delta, theta and lower alpha). To evaluate differences between conditions we conducted pairwise McNemar tests. To test for differences between groups pairwise Chi-square test were applied. Corrections for multiple comparisons were performed according to Benjamini and Hochberg [2].

## Results

In the following, we will present event-related synchronisation/desynchronisation (ERS/ERD), inter-trial (phase locking index, PLI) and inter-electrode phase-locking (phase locking value, PLV) results. We will start by presenting the healthy control results (ANOVAs STIMULUS × TIME for ERS/ERD, PLI and PLV) of the conducted EEG analyses and in a second step will take group differences (ANOVAs GROUP × STIMULUS × TIME for ERS/ERD, PLI and PLV) into account. For an extended results section, please refer to supplement 1.

### Healthy controls

#### ERS/ERD: passive condition

##### Delta

**Fig. 1:**
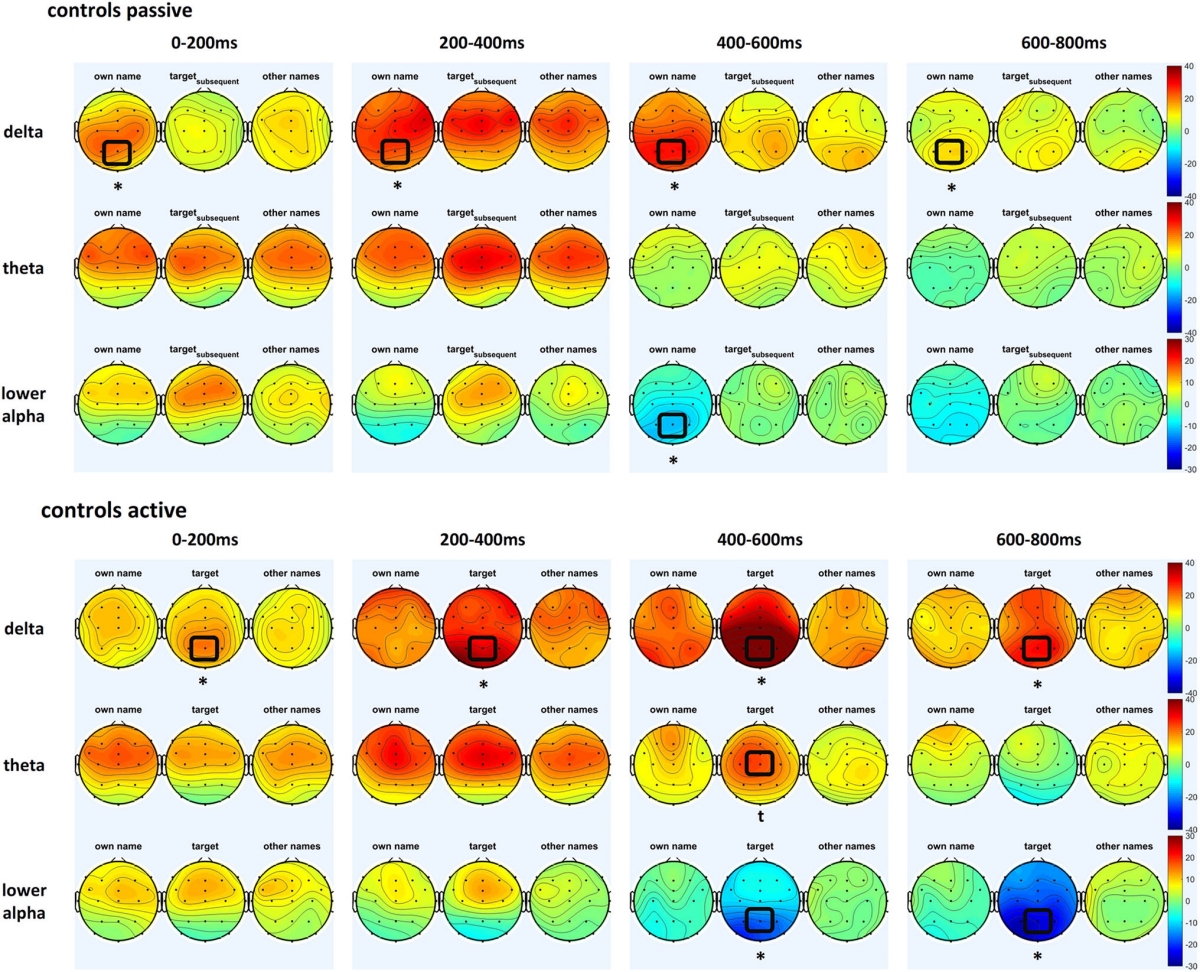
ERS/ERD scalp maps for controls for the three frequency bands, delta, theta and lower alpha. **a** Passive condition: while posterior delta ERS was highest for the own name in all time windows, lower alpha desynchronization indexed the focus of attention only in the third time window from 400 to 600 ms. In the passive condition, theta was not selective for the different stimuli. **b** Active condition: delta ERS and lower alpha ERD were indicative of the attentional focus on the target name. In general, reactivity even seemed more pronounced than in the passive condition. *Rectangles* indicate significantly stronger ERS/ERD for the own as compared to all other names at *p < .05, ^t^ *p* < .10

We found trends toward interactions STIMULUS x TIME on parietal and occipital midline electrodes (Pz: *F*_6,138_ = 1.88, *p* = .088, Oz: *F*_6,138_ = 2.05, *p* = .063) as well as a significant main effect of STIMULUS on electrode Pz: *F*_2,46_ = 3.88, *p* < .05. Post hoc results indicated higher delta ERS for the own name as compared to the other names across all time windows (own name > “later target”; *T*_23_ = 2.62, *p* < .05; own name > all other names: *T*_23_ = 1.97, *p* = .06, cf. Fig. 1) with strongest differences during early time windows.

##### Theta

Theta in the passive condition did not yield any stimulus-specific differences.

##### Lower alpha

The ANOVA revealed significant main effects for STIMULUS at electrode Pz (*F*_2,46_ = 5.82, *p* < .01) as well as a significant interaction STIMULUS × TIME (*F*_6,138_ = 2.37, *p* < .05). Post hoc tests showed that in the time window from 400 to 600 ms alpha ERD was lowest in response to the own name as compared to the later target and all other names (all *T*_23_ > 3.26, *p* < .05).

At Oz, the general activation pattern was similar to the one on Pz.

#### ERS/ERD: active condition

##### Delta

The ANOVA revealed a main effect for STIMULUS (Pz: *F*_2,46_ = 15.53, *p* < .001, Oz: *F*_2,46_ = 11.29, *p* < .001). Post hoc tests indicated that the target elicited higher delta ERS than the other stimuli (*T*_23_ > 3.87, *p* < .05).

##### Theta

**Fig. 2:**
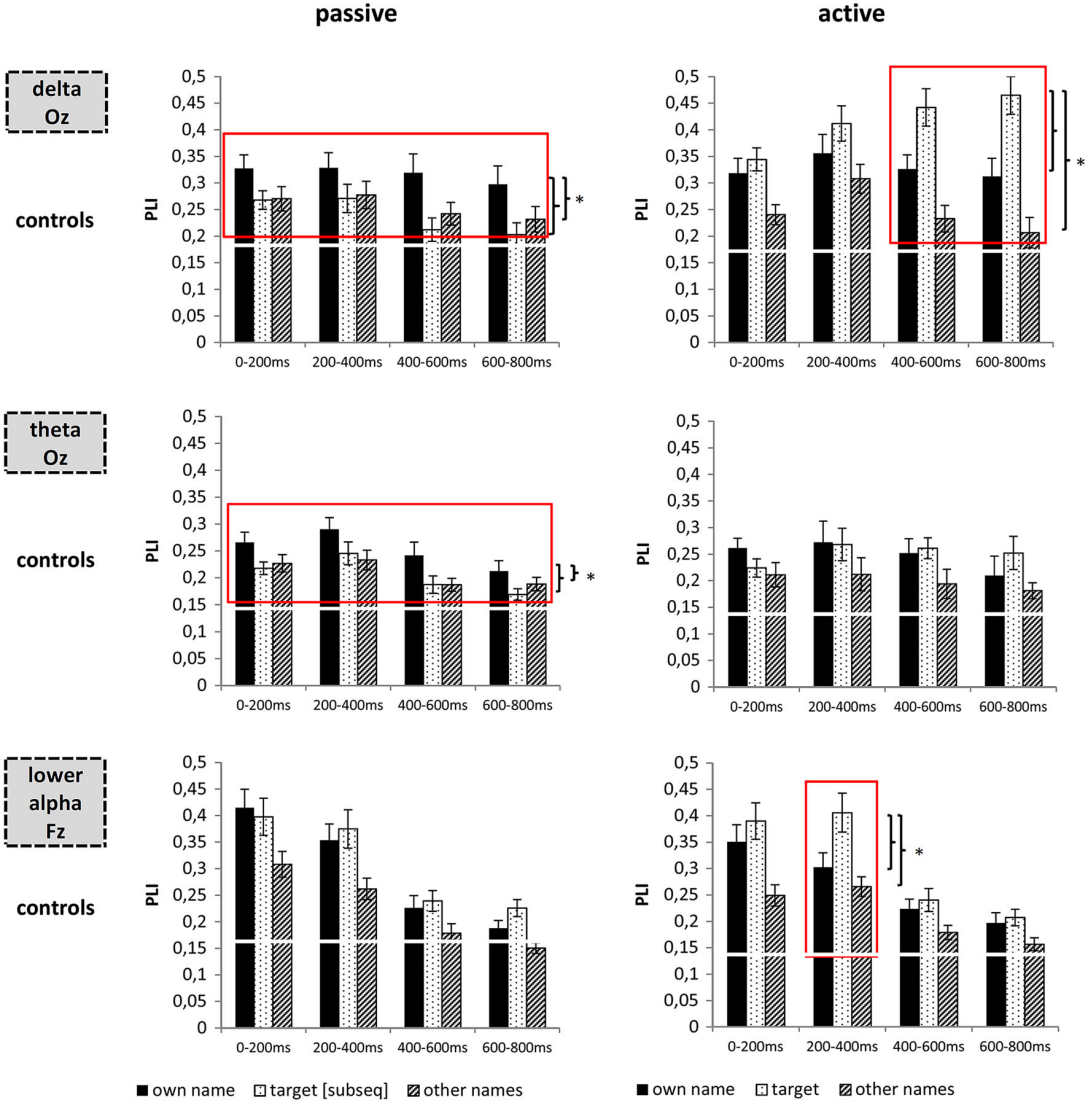
Delta, theta and lower alpha PLI in response to the different stimuli in the passive and the active condition in healthy controls. While delta and theta PLI indicated the focus of attention in the passive condition, delta and lower alpha PLI were pronounced for the target in the active condition. *Red rectangles* indicate the time windows in which the own name showed significantly stronger PLI as compared to all other names, or the target showed higher PLI as compared to both the own and the other names. *p < .05

The ANOVA revealed an interaction between STIMULUS and TIME (Fz: *F*_6,138_ = 2.62, *p* < .01; Cz: *F*_6,138_ = 3.82, *p* < .01). Concerning the interaction, Fig. 2 as well as post hoc tests indicated that in the time window from 400 to 600 ms theta ERS was highest for targets. Post hoc results did, however, not survive the correction for multiple comparisons (target > own name, *T*_23_ = 2.19, *p* = .04, target > other names, *T*_23_ = 2.82, *p* = .01, uncorrected).

##### Lower alpha

The ANOVA at electrode Pz revealed a significant main effect for STIMULUS (*F*_2,46_ = 12.24, *p* < .001) as well as a significant interaction (*F*_6,138_ = 17.93, *p* < .001). Post hoc tests showed that in the two later time windows, ERD was stronger for the target as compared to the own and the other names (*T*_23_ > 3.69, *p* < .05). Results Oz were again very similar to Pz results.

#### PLI: passive condition

##### Delta

ANOVAs for delta PLI for positions Pz and Oz revealed results well comparable to the delta ERS analyses. The ANOVA yielded a significant main effect for STIMULUS (Pz: *F*_2,46_ = 3.95, *p* < .05; Oz: *F*_2,46_ = 5.86, *p* < .01). Post hoc results indicate higher delta PLI for the own name as compared to the other names (Pz, Oz: all *T* > 2.07, all *p* < .05, cf. Fig. 2; all Rayleigh tests for own names—except for Pz time window 400–600 ms—*p* < .05).

##### Theta

All midline electrodes showed main effects for STIMULUS (e.g. Cz: *F*_2,46_ = 6.85, *p* < .01). However, only at Oz, the own name resulted in higher theta inter-trial phase locking as compared to all other names including the later target (*T*_23_ > 2.61, *p* < .05, cf. Fig. 2; Rayleigh test for own names time windows 0–200, 200–400, 600–800 ms *p* < .05, time window 400–600 ms *p* < .10).

##### Lower alpha

All midline electrodes except for Oz showed a main effect for STIMULUS (*F*_2,46_ > 5.23, *p* < .01). The strongest PLI was observed above frontal areas. Concerning the main effect for STIMULUS, the own name and the subsequent target resulted in a higher PLI than the other names (*T*_23_ > 3.20, *p* < .01, cf. Fig. 2, Rayleigh tests for time windows 0–200 and 200–400 ms < .05). The own name and the subsequent target, however, did not differ.

#### PLI: active condition

##### Delta

The ANOVA for electrode positions Pz and Oz revealed a significant main effect for STIMULUS (Pz: *F*_2,46_ = 17.02, *p* < .001; Oz: *F*_2,46_ = 25.94, *p* < .001) as well as an interaction between STIMULUS and TIME (Pz: *F*_6,138_ = 3.39, *p* < .01, Oz: *F*_6,138_ = 3.97, *p* < .01). On Pz and Oz, the target always presented with a higher phase-locking as compared to the other names (Pz: *T*_23_ > 2.47, *p* < .05; Oz: *T*_23_ > 3.64, *p* < .05; Rayleigh tests for target stimuli at both electrodes and all time windows *p* < .05). Phase locking was also higher for the target as compared to the own name on Pz in the last time window (*T*_23_ = 2.52, *p* < .05) and on Oz in the last two time windows (*T*_23_ > 3.15, *p* < .05, cf. Fig. 2).

##### Theta

All midline electrodes showed a main effects for STIMULUS (e.g. Cz: *F*_2,46_ = 5.06, *p* < .05; Oz: *F*_2,46_ = 8.88, *p* < .01). Both the own name and the target unfamiliar name showed higher values as compared to the other names (Cz: *T*_23_ > 2.79, *p* < .05, Oz: *T*_23_ > 3.71, *p* < .01; Rayleigh tests for own names and targets at both electrodes in all time windows *p* < .05, except for own name at Pz time window 400–600 ms: *p* < .10) the own name and the target did, however, not differ (cf. Fig. 2).

##### Lower alpha

ANOVAs revealed a main effect for STIMULUS (*F*_2,46_ > 5.98, *p* < .05) as well as an interaction between STIMULUS and TIME (*F*_6,138_ > 2.65, *p* < .05) at all midline electrodes. Post hoc tests for electrode Fz showed that overall both the own name and the target elicited a stronger phase locking than the other names (*T*_23_ > 3.00, *p* < .05; Rayleigh tests for own names and target in time windows 0–200 and 200–400 ms *p* < .05). Testing the interaction between TIME and STIMULUS, post hoc results for Fz additionally revealed that in the second time window from 200 to 400 ms PLI was higher for the target as compared to both the own name and the other names (cf. Fig. 2).

#### PLV: passive condition

##### Delta

**Fig. 3:**
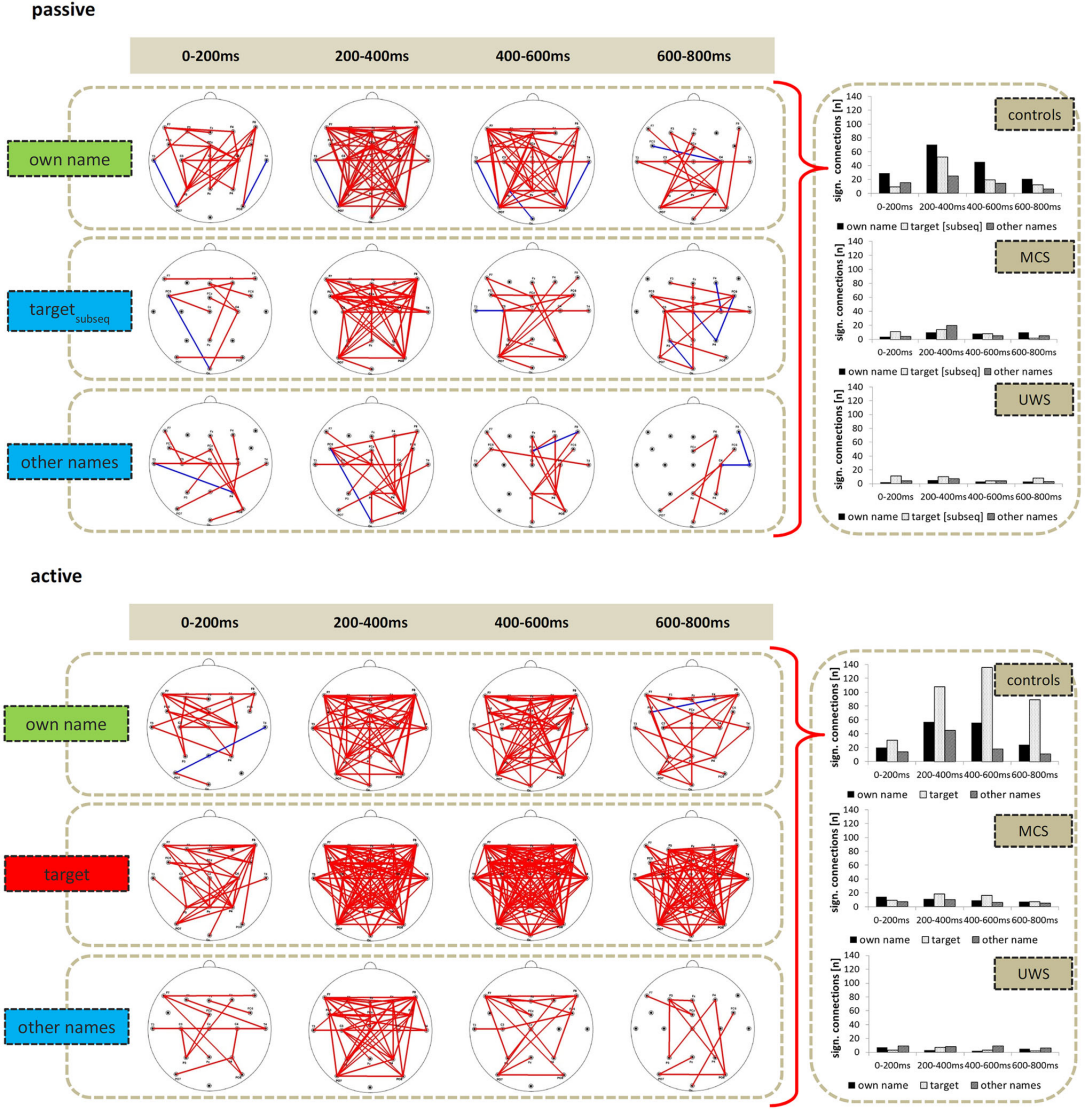
Number of significant delta PLV connections as revealed by permuted *t* tests against baseline. Overall controls showed higher network density as compared to patients. Furthermore, in controls the number of connections was highest for the own name in the passive and for the target in the active condition. Within patient groups, no stimulus-specific differences in any of the time windows could be observed

In the first three time windows, i.e., from 0 to 600 ms, the own name always elicited a more densely connected network as represented by more connections with a significantly higher PLV compared to the baseline interval (McNemar exact *p* < .05, corrected for multiple comparisons, cf. Fig. 3).

Theta and lower alpha PLV did not yield stimulus-specific differences in network density.

#### PLV: active condition

In the later three time windows from 200 to 800 ms the target name always elicited a more densely connected network as compared to both the own name and the other names. In the two later time windows from 400 to 800 ms the presentation of the own name still activated a stronger network as compared to the other names (McNemar exact *p* < .05, cf. Fig. 3). As in the passive condition, theta and lower alpha PLV did not yield stimulus-specific differences in network density.

### Group differences

For reasons of conciseness, only results which indicate group differences will be reported. For extended results, please again refer to the supplementary material.

#### ERS/ERD: passive condition

##### Delta

**Fig. 4:**
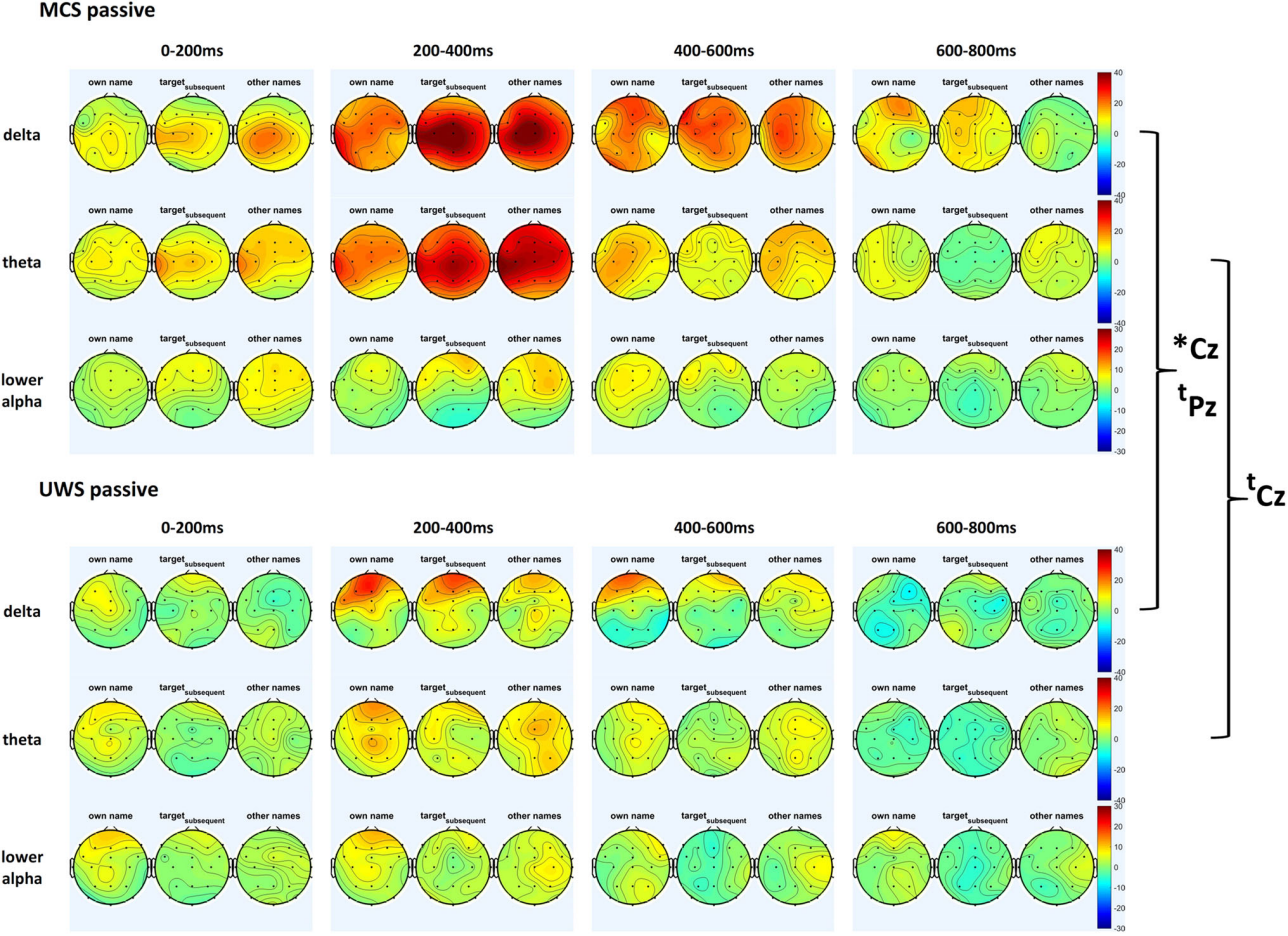
ERS/ERD scalp maps for MCS and UWS patients for the delta, theta and lower alpha band for the passive condition. General delta and theta ERS toward any auditory stimulation differentiated at least by tendency between MCS and UWS patients. *Brackets* indicate stronger ERS in MCS as compared to UWS patients at *p < .05 and ^t^ *p* < .10

Electrodes Cz, Pz and Oz showed a main effect for GROUP (Cz: *F*_2,36_ = 4.11, *p* < .05; Pz: *F*_2,36_ = 5.37, *p* < .01; Pz: *F*_2,36_ = 4.61, *p* < .01). Post hoc tests revealed no significant differences between controls and MCS patients. UWS patients exhibited significantly weaker delta ERS than controls (all *T*_30_ at Cz, Pz, Oz > 2.91, *p* < .01) and MCS patients (Cz: *T*_13_ = 2.17, *p* < .05 and Pz: *T*_6.86_ = 2.20, *p* = .064 cf. Fig. 4).

##### Theta

The ANOVA did not reveal group differences. As this could have resulted from the similarity between controls and MCS patients, we compared theta ERS at Fz and Cz between groups. Indeed, results indicated a tendency toward higher ERS in controls and MCS patients as compared to UWS patients (Fz: *T*_30_ = 2.01, *p* = .06 and *T*_13_ = 1.77, *p* = .10; Cz: *T*_30_ = 1.94, *p* = .06 and *T*_13_ = 2.08, *p* = .06, cf. Fig. 4).

##### Lower alpha

Analyses at electrode Pz revealed an interaction STIMULUS × GROUP (*F*_4,72_ = 3.27, *p* < .05). Post hoc test showed, that again only controls but not patients showed a differential response to the own name and presented with significantly stronger lower alpha ERD for the own name as compared to the later target and the other names (*T*_23_ > 2.68, *p* < .05).

#### ERS/ERD: active condition

##### Delta

The ANOVA for position Cz revealed a main effect for GROUP (*F*_2,36_ = 5.62, *p* < .01) as well as interactions between GROUP × TIME (*F*_6,108_ = 2.20, *p* < .05) and GROUP × STIMULUS (*F*_6,72_ = 2.53, *p* < .05). Analyses at Pz and Oz revealed a similar picture.

In general, controls showed higher delta ERS than UWS patients at all electrodes (*T*_30_ > 2.69, *p* < .05). When compared to MCS, controls showed stronger ERS at Oz (*T*_29_ = 2.89, *p* < .05) and, by tendency, also at Fz (*T*_29_ = 1.88, *p* = .071). Comparing the two patient groups, delta ERS was, by tendency, stronger in MCS than in UWS patients (Pz: *T*_7.13_ = 1.91, *p* = .098; Oz: *T*_13_ = 1.871, *p* = .084, cf. supplementary figure 2). Analyses did not reveal any stimulus-specific differences (own name vs. target vs. other names) in MCS or in UWS patients.

##### Theta

The ANOVA at position Cz indicated a trend towards a main effect for GROUP (*F*_2,36_ = 3.06, *p* = .06; cf. Fig. 2). Post hoc comparisons only revealed that controls showed overall higher theta ERS than both MCS (tendency at *T*_28.17_ = 1.95, *p* = .06) and UWS (*T*_28.13_ = 3.17, *p* < .01) patients.

##### Lower alpha

The ANOVA for position Oz yielded a significant interaction between GROUP and TIME (*F*_6,108_ = 3.39, *p* < .01, cf. supplement 1). Post hoc tests indicated that in the last time window from 600 to 800 ms lower alpha ERD was strongest in controls compared to MCS (*T*_29_ = −3.08, *p* < .05) and UWS (*T*_29_ = −3.93, *p* < .05) patients. No differences between MCS and UWS were evident in the lower alpha band.

#### PLI: passive condition

##### Delta

The ANOVA for position Pz indicated a main effect of GROUP (*F*_2,36_ = 5.44, *p* < .01). According to post hoc results controls showed higher PLI values as compared to UWS (*T*_30_ = 4.75, *p* < .01) patients. MCS patients did not differ from UWS patients. The same analysis for position Oz showed a similar picture.

##### Theta

The ANOVA yielded a main effect of GROUP (Cz: *F*_2,36_ = 10.01, *p* < .001; Oz: *F*_2,36_ = 4.27, *p* < .05). Overall, controls presented with higher theta PLI as compared to UWS patients (Cz: *T*_30_ = 4.96, *p* < .001; Oz: *T*_30_ = 3.67, *p* < .01). When compared to MCS, controls only showed a trend towards higher theta PLI at electrode Cz (*T*_29_ = 2.04, *p* = .051).

##### Lower alpha

The ANOVA for position Fz revealed a main effect of GROUP (*F*_2,36_ = 13.63, *p* < .001). Controls showed higher PLI values as compared to both UWS (*T*_30_ = 4.67, *p* < .01) and MCS patients (*T*_29_ = 3.75, *p* < .01).

#### PLI: active condition

##### Delta

The ANOVA at position Pz revealed a main effect for GROUP (*F*_2,36_ = 6.56, *p* < .01). Post hoc tests revealed higher PLI values in controls than in UWS (*T*_30_ = 4.97, *p* < .001) but not than in MCS patients. Results for Oz were again similar.

##### Theta

The ANOVA revealed a main effect of GROUP (Cz: *F*_2,36_ = 8.32, *p* < .01; Oz: *F*_2,36_ = 6.78, *p* < .01). Post hoc tests indicated higher PLI values in controls as compared to UWS (Cz: *T*_30_ = 4.65, *p* < .001; Oz: *T*_30_ = 4.45, *p* < .001). Interestingly, comparing the two patient groups, on Oz MCS patients also showed by tendency higher theta PLI when compared to UWS (*T*_13_ = 2.07, *p* = .06).

##### Lower alpha

The ANOVA for position Fz revealed a main effect of GROUP (*F*_2,36_ = 12.39, *p* < .001). Post hoc test revealed higher PLI values in controls as compared to both UWS (*T*_30_ = 4.75, *p* < .001) and MCS (*T*_29_ = 3.26, *p* < .01) patients.

#### PLV: passive condition

Chi-square tests indicated a higher network density in controls as compared to both MCS and UWS patients in response to the own name. HC vs. MCS: the number of significant connections was higher in the three time windows from 0 to 600 ms; HC vs. UWS: the number of connections was higher in all four time windows (*χ*^2^ > 13.31, *p* < .05). Between patient groups, only on one occasion network density was higher for MCS than for UWS patients (other names, 200 to 400 ms, *χ*^2^ = 6.55, *p* < .05).

Within patient groups McNemar tests did not reveal significant differences between stimuli in any of the four time windows.

Since theta and lower alpha did not reveal stimulus-specific differences in network connectivity in controls, they were not subjected to a group analysis.

#### PLV: active condition

Chi-square tests again revealed higher network density in controls as compared to both MCS and UWS patients in response to the target (all *χ*^2^ > 13.70, *p* < .05) and the own name (as compared to MCS in the three time windows from 200 to 800 ms and as compared to UWS in all four time windows, *χ*^2^ > 6.56, *p* < .05). Between patient groups, again, on one occasion network density for the target name was higher for MCS as compared to UWS patients (400–600 ms, *χ*^2^ = 9.42, *p* < .05).

Within patient groups McNemar tests did not reveal significant differences between stimuli in any of the four time windows.

Again, since theta and lower alpha did not reveal stimulus-specific differences in network connectivity in controls, theta and lower alpha connections were not subjected to a group analyses.

## Discussion

Several studies have used the auditory own name paradigm in order to test differential stimulus processing in states with impaired consciousness such as sleep or DOC [34, 35, 39]. Our aim was to contribute to this line of research by focussing on time and non-time-locked local oscillatory EEG activity in the delta, theta and lower alpha band to have a more sensitive analysis in a group of severely brain injured patients, in whom also the timing of brain responses might be affected. Additionally, we calculated phase-locking between electrode sites to get an estimate of connectivity between scalp sites.

In controls, delta ERS and lower-alpha ERD nicely indicated the focus of attention in both the passive as well as the active condition (cf. Fig. 1). While delta ERS and lower-alpha ERD were strongest for the own name in the passive condition, where the own name has been shown to automatically catch attention [32, 49], the same oscillations were strongest for the target in the active condition, in which participants had been asked to wilfully shift their attention. Topographically, both effects were present on parietal to occipital midline sites. As both delta and lower alpha power have previously been related to general attention [16, 19, 23] our results may indicate a sensitivity to both automatic attention capture by the own name in the passive condition as well as to specific top–down attentional demands by the instruction to silently count the target name in the active condition. In contrast to the increase in delta power, alpha desynchronized in late time windows, which should be related to the release from inhibition in the respective networks [22].

Furthermore, in the active but not in the passive condition also late theta ERS reflected the voluntary attention shift, i.e., theta ERS was increased at 400–600 ms after stimulus onset for the target name, which had to be counted silently, with a dominance at fronto-central midline sites. This increase in theta ERS towards target stimuli is well in line with previous findings that frontal theta band power and phase synchronisation are related to working memory processes (for a review, please refer to [23, 37]) which seem to be required for memorising the task instruction and keeping count of the number of target appearances. Theta increase to target names has also been shown in healthy in the study by Fellinger et al. [12].

We aimed at complementing the event-related power analyses by additional inter-trial and inter-electrode phase-locking calculations to test, if also local timing and network density changed in response to different stimuli and task demands. In controls, occipital delta inter-trial phase coupling again indexed the focus of attention in both conditions similarly to delta ERS. Previously, it has been shown that the phase of slower oscillations synchronises or modulates the amplitude of higher oscillations [4, 21, 29]. Especially in the active condition, where attentional demands were higher, the time course of delta phase locking both between trials and between electrodes strongly resembled the time courses of alpha ERD, which could hint at a cross-link of delta and alpha oscillations in this task. In the theta and alpha bands, phase locking results were not as indicative as the ERS/ERD analysis. Theta PLI indicated the focus of attention only in the passive condition. Alpha PLI, on the other hand, was only in the active condition higher for the target as compared to the own and other names. This effect was, however, shifted from occipital to frontal regions. While alpha ERD peaked in the late time windows, the increase in frontal alpha PLI was present in the early time windows. The early alpha PLI increase might relate to the access of semantic memory triggered by bottom–up (passive condition) or top–down (active condition) attentional modulation, similarly as has been shown for the early P1 component [50], which has been linked to the re-alignment of ongoing alpha activity [15].

With regard to inter-regional phase coupling, a widespread delta network differentiated between (automatically) attended and unattended stimuli (cf. Fig. 3). This is in line with the notion that long-range information transfer is established by synchronisation in low frequencies [47, 48]. The fact that the delta network was topographically largely extended could indicate a role of delta frequency for the large-scale integration into a global workspace, which would in turn point at a relationship between delta connectivity and consciousness.

In patients, we have not found stimulus-specific differences at group level, while other groups using EEG or fMRI in combination with auditory stimulation have reported such [6, 35, 39, 43]. Theta ERS has previously been shown to even increase for target stimuli in MCS, but not in UWS patients [12]. This difference could only be observed, however, when the target was the subject’s own name and not when it was an unfamiliar name. In our study, the target was an unfamiliar name and did not produce a consistent increase in theta power in MCS patients. This could indicate that patients had problems to allocate attention to a stimulus that does not contain sufficient bottom–up strength such as the own name. However, the fact that in even in the passive condition we did not see clear differences between stimuli in MCS limits this interpretation.

Generally, event-related increases in delta and theta power independent of the type of stimulus or task condition, however, proved useful in order to differentiate between MCS and UWS patients. In the active condition, even theta inter-trial (main effect at Oz) and delta inter-electrode phase locking (for targets at 400–600 ms) seemed to be (by tendency) higher in MCS than UWS patients. It has been claimed earlier that active task demands should be necessary in order to differentiate MCS from UWS, e.g., in the auditory own name paradigm [39]. Our results, however, do not support this assumption. Together with findings questioning the reliability of active task performances even in healthy individuals [9, 18], we believe that a clinically reliable measure should index a patient’s conscious state on multiple levels including those which are independent of task demands such as instruction following or other complex abilities. Promising approaches might include resting state analyses [28, 42, 46] or measures like the Perturbational Complexity Index [5, 30, 33]. Also, Sitt et al. [41] have in a large-scale study involving 173 MCS and UWS patients recently demonstrated, that low frequency power (delta, theta, alpha) as well as complexity and information transfer measures derived from the EEG are the most reliable measures to distinguish MCS from UWS during auditory stimulation independently of the type of stimulus. In contrast to the study by Sitt et al., our results also indicate, that not only power, but also frequency band specific reactivity (i.e., ERS/ERD) could be useful for differentiating MCS from UWS.

It is important to note here, that also UWS showed clearly discernible stimulus related increases in the delta and theta band. However, except for a visible increase in delta PLI towards the own name, UWS did mostly not show a clear even-related increase in inter-trial phase-locking as compared to baseline. This indicates an impaired timing of brain processing with decreased consciousness level. This finding certainly holds important implications for ERP studies in DOC, which do study strictly time-locked (“evoked”) activity and might, therefore, miss stimulus-specific brain responses, which are not as strictly time-locked but “induced oscillations”. As a consequence, we propose to not limit EEG analyses to time-locked activity especially when analysing DOC data, but to amend this approach by separating induced and evoked responses, as exact and unaltered timing of processing is unlikely given the severe impairments in DOC.

## Conclusion

While controls presented with systematic changes in event-related delta, theta and lower alpha oscillations, phase-realignment and inter-electrode connectivity in response to salient bottom–up (own name) or top–down (attended) targets, the studied DOC patients did not. On the other hand, patients in MCS could be differentiated from UWS patients based on their general (independent of the types of stimuli) event-related power increases in the delta and (by tendency) theta band. Results suggest that to gain a comprehensive picture of patients’ capabilities, these patients should be evaluated across a range of methods focusing on evoked (ERP) as well as induced activity in the EEG and include connectivity measures. With respect to cognitive paradigms, we favour a hierarchical approach also including measures of brain activity independent of task demands which do not rely on higher-order functions such as language comprehension and intact working memory capacity.

## Electronic supplementary material

Below is the link to the electronic supplementary material.

Supplementary material 1 (TIFF 16167 kb)

Supplementary material 2 (TIFF 16295 kb)

Supplementary material 3 (TIFF 16796 kb)

Supplementary material 4 (TIFF 15948 kb)

Supplementary material 5 (DOCX 37 kb)

Supplementary material 6 (DOCX 15 kb)
