# Supplementary figures and images for: Event-related EEG power modulations and phase connectivity indicate the focus of attention in an auditory own name paradigm
Source: J Neurol. 2016 May 23;263:1530–43. doi: 10.1007/s00415-016-8150-z (PMC4971049; doi:10.1007/s00415-016-8150-z)

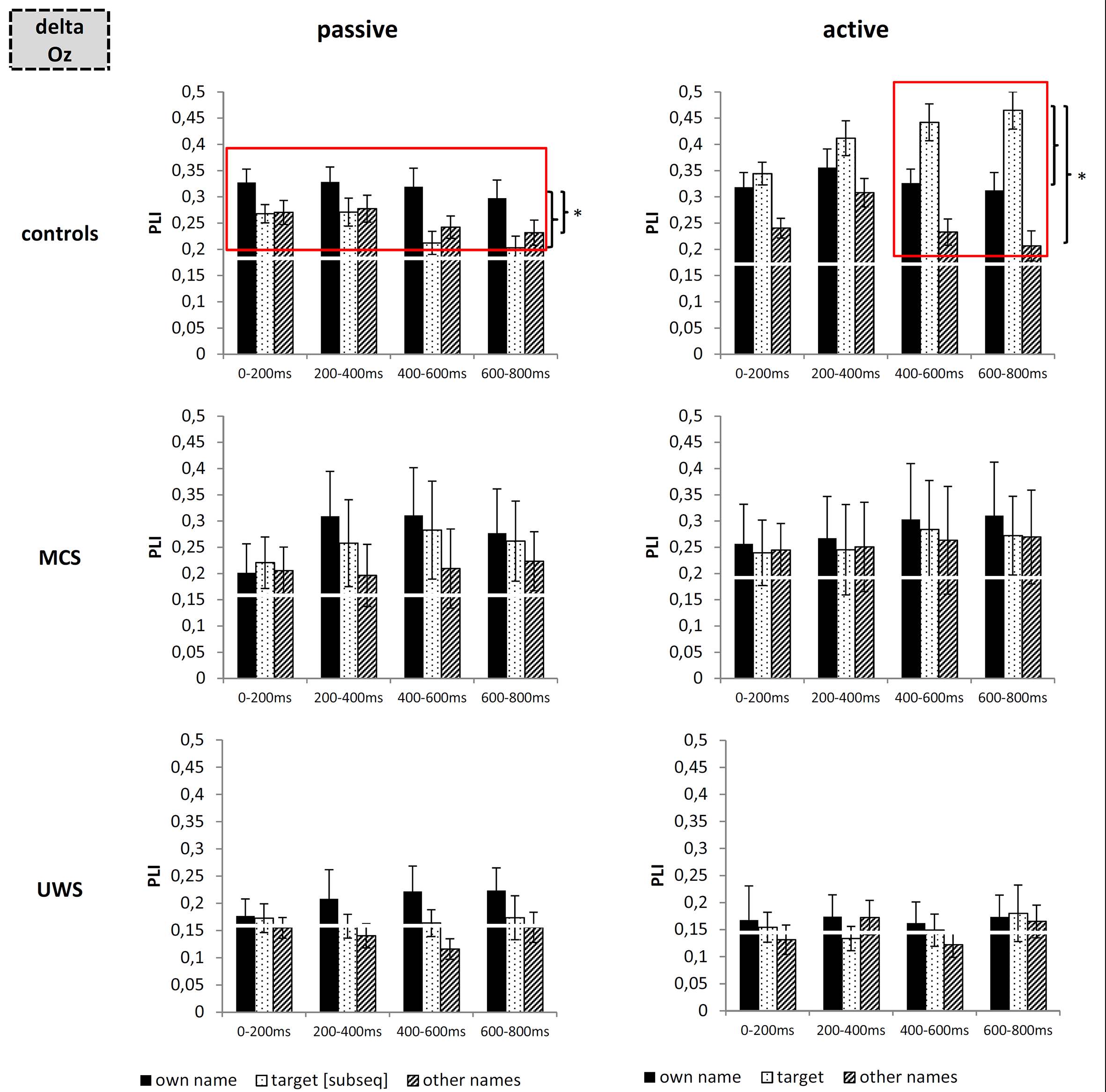

Supplement: Supplementary file 1 — Supplementary material 1 (TIFF 16167 kb) [file 415_2016_8150_MOESM1_ESM.tif]

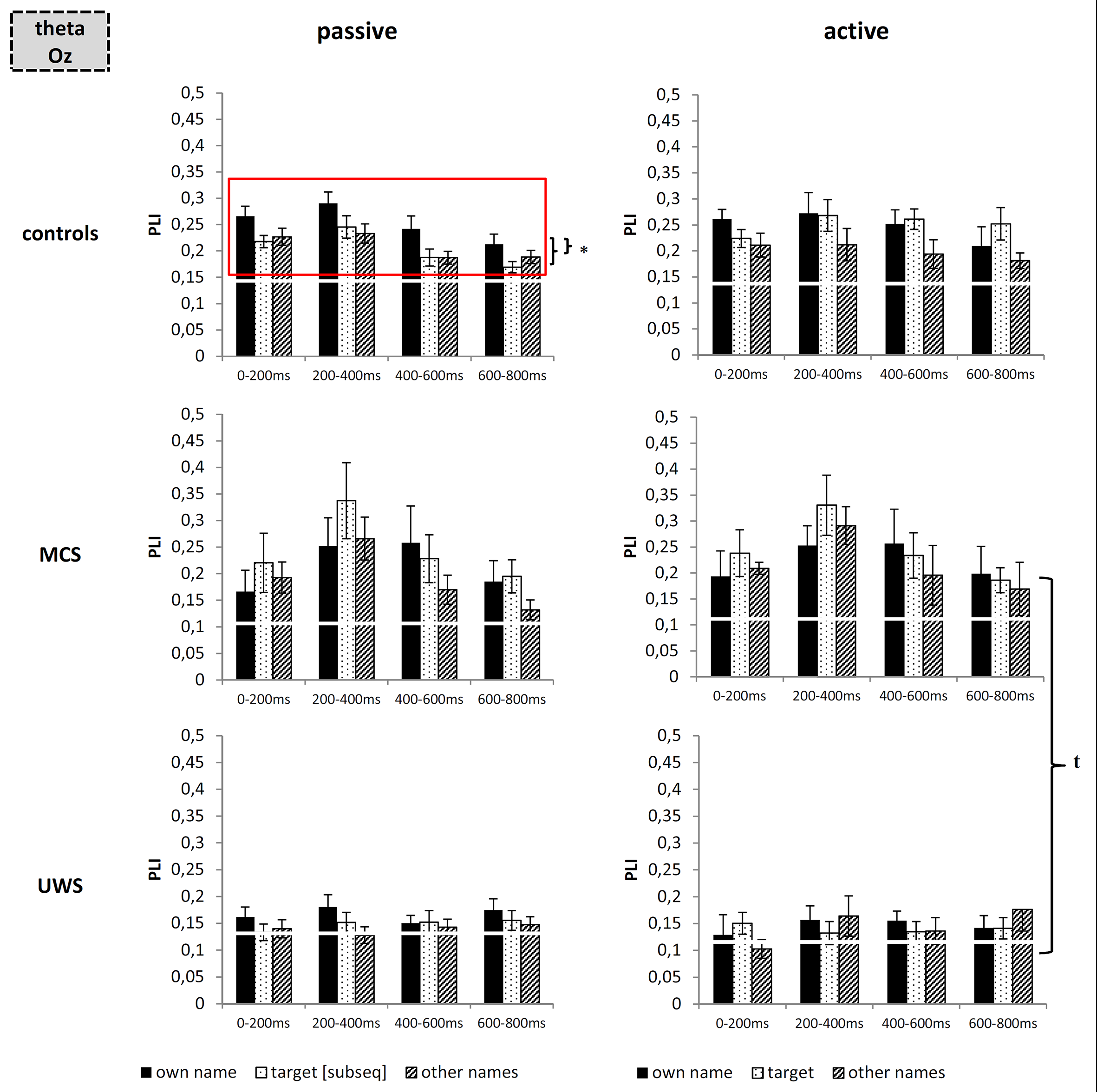

Supplement: Supplementary file 2 — Supplementary material 2 (TIFF 16295 kb) [file 415_2016_8150_MOESM2_ESM.tif]

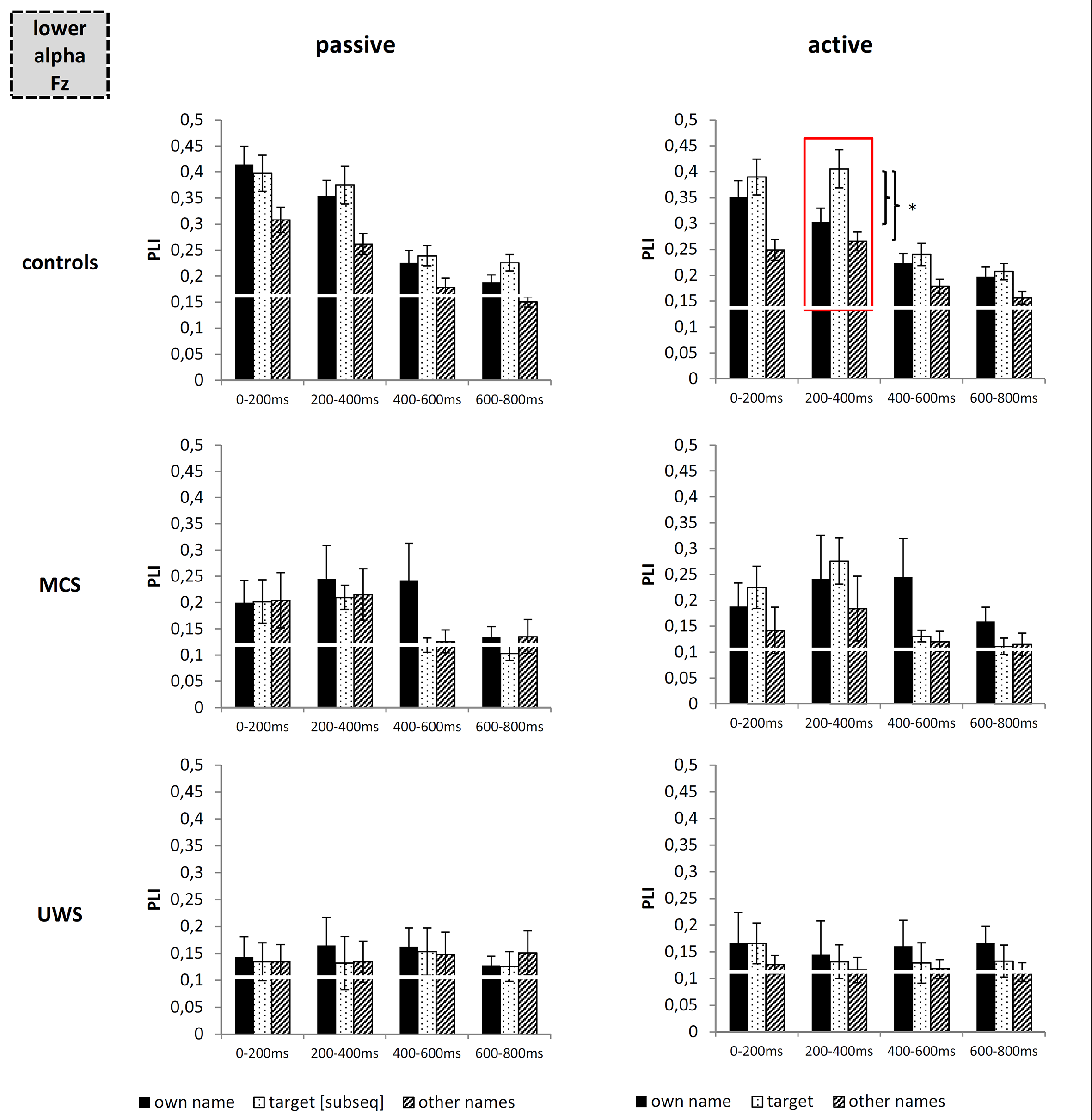

Supplement: Supplementary file 3 — Supplementary material 3 (TIFF 16796 kb) [file 415_2016_8150_MOESM3_ESM.tif]

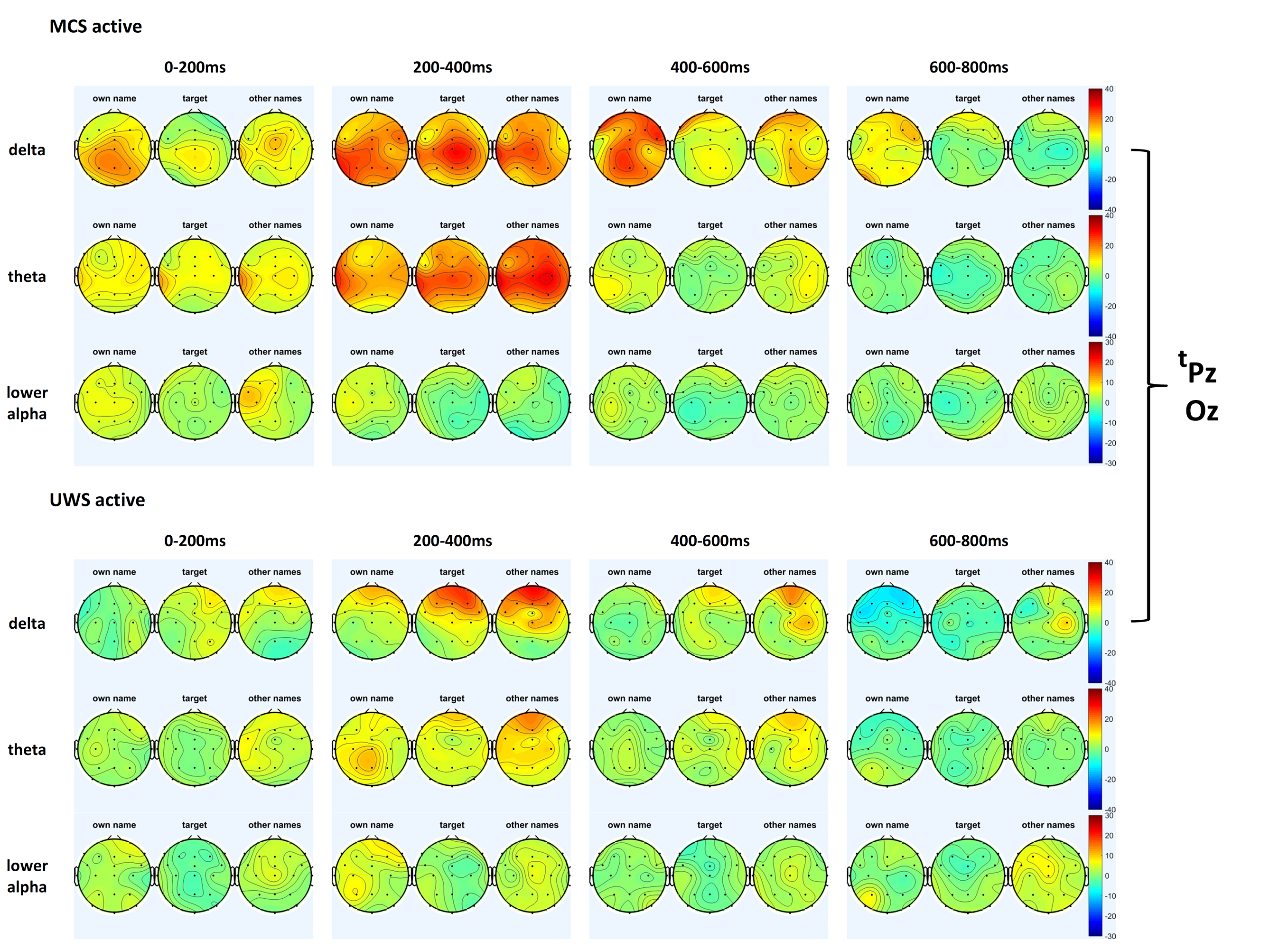

Supplement: Supplementary file 4 — Supplementary material 4 (TIFF 15948 kb) [file 415_2016_8150_MOESM4_ESM.tif]
