# Supplementary material for: Event-related EEG power modulations and phase connectivity indicate the focus of attention in an auditory own name paradigm
Source: J Neurol. 2016 May 23;263:1530–43. doi: 10.1007/s00415-016-8150-z (PMC4971049; doi:10.1007/s00415-016-8150-z)
Supplement: Supplementary file 5 — Supplementary material 5 (DOCX 37 kb) [file 415_2016_8150_MOESM5_ESM.docx]

**Event-related EEG power modulations and phase connectivity indicate the focus of attention in an auditory own name paradigm**

Lechinger, Julia^1,2,^*

Wielek, Tomasz^1^

Blume, Christine^1,2^

Pichler, Gerald^3^

Michitsch, Gabriele^4^

Donis, Johann^4^

Gruber, Walter^2^

Schabus, Manuel^1,2^

Author Affiliation:

^1^Laboratory for Sleep and Consciousness Research, University of Salzburg, Austria

^2^Centre for Cognitive Neuroscience (CCNS), University of Salzburg, Austria

^3^Apallic Care Unit, Neurological Division, Albert-Schweitzer-Klinik, Graz, Austria

^4^Apallic Care Unit, Neurological Division, Sozialmedizinisches Zentrum Ost - Donauspital, Vienna, Austria

*Corresponding author

University of Salzburg

Department of Psychology

Laboratory for Sleep, Cognition and Consciousness

Hellbrunnerstraße 34

5020 Salzburg, Austria

e-mail: [julia.lechinger@sbg.ac.at](mailto:julia.lechinger@sbg.ac.at)

**Supplementary results**

In the following, we report extended results. The structure and the numbering of the paragraphs follow the results section in the main document.

**3. Results**

In the following, we will present ERS/ERD, inter-trial (PLI) and inter-electrode phase-locking (PLV) results. We will start by presenting the healthy control results of the conducted EEG analyses and in a second step will take group differences into account.

3.1 Healthy controls

3.1.1 ERS/ERD: passive condition

*Delta*

Delta ERS was most prominent above parietal and occipital areas, wherefore we will only report Pz and Oz results. Calculating ANOVAs for STIMULUS x TIME, both posterior midline electrodes showed a significant main effect for TIME (Pz: F_3,69_ = 9.39, p < .001, Oz: F_3,69_ = 4.6, p < .01) and marginally significant interactions STIMULUS x TIME (Pz: F_6,138_ = 1.88, p =.088, Oz: F_6,138_ = 2.05, p =.063). Furthermore, the parietal midline electrode also showed a significant main effect of STIMULUS (Pz: F_2,46_ = 3.88, p < .05). Post-hoc results indicated higher delta ERS for the own name as compared to the other names across all time windows (own name > “later target”; T_23_ = 2.62, p < .05; own name > all other names: T_23_ = 1.97, p = .06). Although the interaction with TIME was not significant, strongest differences between the stimuli were obvious in the earlier time windows. Concerning the main effect for TIME, post-hoc tests for Pz revealed, that intermediate time windows showed higher ERS than the first (0-200ms) and the last (600-800ms) time window (all |T_23_| > 2.70, p < .05). On Oz, only the time window from 400-600ms showed stronger ERS than the early time window from 0-200ms (T_23_ = 3.50, p < .05).

*Theta*

Theta ERS was most prominent above frontal and central sites. The ANOVA STIMULUS x TIME at electrode only revealed a main effect for time (Fz: F_3,69_ = 38.29, p < .001; Cz: F_3,69_ = 34.27, p < .001). Overall, the early time window from 0-200ms and 200-400ms showed stronger theta ERS than the later time windows from 400-600ms and 600-800ms (T_23_ > 4.58, p < .001). Furthermore, ERS was still higher from 400-600ms as compared to 600-800ms (T_23_ > 3.80, p < .01).

*Lower Alpha*

Lower alpha ERD was strongest at parietal and occipital sites. The ANOVA STIMULUS x TIME for electrode Pz revealed significant main effects for STIMULUS (F_2,46_ = 5.82, p < .01) and TIME (F_3,69_ = 9.56, p < .001) as well as a significant interaction (F_6,138_ = 2.37, p < .05). Post-hoc tests showed that in the time window from 400-600ms alpha ERD was lowest in response to the own name as compared to the later target and all other names (all T_23_ > 3.26, p < .05). Within the response to the own name, ERD was stronger from 400-600ms as compared to both earlier time windows (T_23_ > 3.41, p < .05).

At Oz, the general activation pattern was similar to the one on Pz, the ANOVA STIMULUS x TIME, however, only showed a significant main effect for STIMULUS (F_2,46_ = 5.67, p < .01).

3.1.2 ERS/ERD: active condition

*Delta*

Delta ERS was again strongest at parieto-occipital sites. The ANOVA revealed main effects for both TIME (Pz: F_3,69_ = 13.24, p < .001, Oz: F_3,69_ = 23.53, p < .001) and STIMULUS (Pz: F_2,46_ = 15.53, p < .001, Oz: F_2,46_ = 11.29, p < .001) as well as the interaction between TIME and STIMULUS (Pz: F_6,138_ = 6.92, p < .001, Oz: F_6,138_ = 7.81, p < .001). Post-hoc tests indicated that on both electrodes delta ERS was highest in the third time window from 400-600ms as compared to all other time windows (T_23_ > 3.88, p < .05). Concerning the main effect for STIMULUS, the target elicited higher delta ERS than the other stimuli (T_23_ < 3.87, p < .05)

*Theta*

The ANOVA STIMULUS x TIME at electrode Fz and Cz again revealed a main effects for time (Fz: F_3,69_ = 19.87, p < .001; Cz: F_3,69_ = 20.96, p < .001) as well as an interaction between STIMULUS and TIME (Fz: F_6,138_ = 2.62, p < .01; Cz: F_6,138_ = 3.82, p < .01). At Fz, the second time window showed higher ERS than then the later two time windows and in general, the last time window showed the lowest ERS as compared to all other time window (T_23_ > 4.81, p < .05). At Cz, except for the comparison between 200-400ms and 400-600ms, the early time window from 0-200ms and 200-400ms showed a stronger theta ERS than the later time windows from 400-600ms and 600-800ms (T_23_ > 5.12, p < .05). Furthermore, ERS was also higher in the second time window from 200-400ms as compared to the first time window from 0-200ms (T_23_ = 2.27, p < .05). Concerning the interaction, post-hoc tests indicated that in the time window from 400-600ms theta ERS was highest for targets. Post-hoc results did, however, not survive the correction for multiple comparisons (target > own name, T_23_ = 2.19, p = .04, target > other names, T_23_ = 2.82, p = .01, uncorrected).

*Lower Alpha*

The ANOVA STIMULUS x TIME at electrode Pz revealed significant main effects for STIMULUS (F_2,46_ = 12.24, p < .001) and TIME (F_3,69_ = 20.91, p < .001) as well as a significant interaction (F_6,138_ = 17.93, p < .001). Post-hoc tests showed that in the two later time windows, ERD was stronger for the target as compared to the own and the other names (T_23_ > 3.69, p < .05). Furthermore, for both the target as well as the own name ERD was stronger in the two latter time windows (from 400-600ms and 600-800ms) as compared to the two earlier time windows (from 0-200 and 200-400ms) and ERD at 600-800ms was again even stronger than at 400-600ms (T_23_ > 3.69, p < .05, corrected). Results Oz were again very similar to Pz results (STIMULUS: F_2,46_ = 16.76, p < .001; TIME: F_3,69_ = 12.25, p < .001; STIMULUS x TIME: F_6,138_ = 9.79, p < .001).

3.1.3 PLI: passive condition

*Delta*

Although delta inter-trial phase locking (PLI) was slightly stronger above frontal regions, the effect was only stimulus-specific above posterior regions.

ANOVAs for delta PLI for positions Pz and Oz revealed results well-comparable to the delta ERS analyses. The ANOVA STIMULUS x TIME, for electrode positions Pz and Oz revealed significant main effects for TIME (Pz: F_3,69_ = 11.39, p < .001, Oz: F_3,69_ = 4.86, p < .01) and STIMULUS (Pz: F_2,46_ = 3.95, p < .05; Oz: F_2,46_ = 5.86, p < .01). Post-hoc results indicate higher delta PLI across all time windows for the own name as compared to the other names (Pz: own name > “later target”; T_23_ = 2.07, p < .05; own name > all other names: T_23_ = 2.28, p < .05; Oz: own name > “later target”; T_23_ = 2.84, p < .01; own name > all other names: T_23_ = 2.19, p < .05). Concerning the main effect for TIME, post-hoc tests for Pz revealed that the PLI in the first two time windows (0-200ms, 200-400ms) was higher than the PLI in the later two time windows (400-600ms and 600-800ms) (T_23_ > 2.31, p < .05). The same was true for Oz (T_23_ > 2.38, p < .05) except for the comparison between 200-400ms vs. 400-600ms.

*Theta*

Theta PLI was again subjected to ANOVAs with the factors STIMULUS and TIME. All midline electrodes showed a main effects for STIMULUS (e.g. Cz: F_2,46_ = 6.85, p < .01) and TIME (F_3,69_ = 78.75, p < .001). Post-hoc tests concerning the main effect for TIME showed that the time window from 200-400ms presented with the highest theta PLI as compared to all other time windows (T_23_ > 4.45, p < .01). Also, the latest time window from 600-800ms exhibited the lowest theta PLI as compared to all other time windows (T_23_ > 3.39, p < .01). Concerning the main effect for the factor STIMULUS, both the own name and the target_subsequent_ caused higher theta PLI than the other names (T_23_ > 3.12, p < .01) at electrodes Fz, Cz and Pz, whereas the PLI to the own name was *not* higher than the PLI for the later target, suggesting that theta PLI on frontal to parietal positions does not indicate the focus of attention. On Oz, however, the own name resulted in higher theta inter-trial phase locking as compared to all other names (T_23_ > 2.61, p < .05).

*Lower Alpha*

Lower alpha PLI results were very similar to theta PLI results. All midline electrodes except for Oz showed a main effect for STIMULUS (F_2,46_ > 5.23, p < .01). All midline electrodes presented with a main effect of TIME (F_3,69_ > 4.68, p < .001). In contrast to lower alpha PLI, the strongest PLI was not observed above parieto-occipital, but frontal areas. Additionally, while lower alpha PLI increased in later time windows, phase-locking was highest in the earlier time windows. Post-hoc tests for Fz showed that independent of the type of stimulus, the PLI was different between all time windows, with the earlier time window generally being characterised by a higher PLI than the later time window (T_23_ > 2.50, p < .05). Concerning the main effect for STIMULUS, the own name and the subsequent target resulted in a higher PLI than the other names (T_23_ > 3.20, p < .01). The own name and the subsequent target, however, did not differ, i.e. in the passive condition lower alpha PLI seems to be more sensitive to the physical properties than to the saliency of the stimuli.

3.1.4 PLI: active condition

*Delta*

In contrast to the passive condition, the time course of the delta PLI did not closely follow the time course of delta ERS, but rather the one of the lower alpha ERD (see lower alpha ERD results below). The ANOVA STIMULUS x TIME, for electrode positions Pz and Oz revealed significant main effects for STIMULUS (Pz: F_2,46_ = 17.02, p < .001; Oz: F_2,46_ = 25.94, p < .001) as well as an interaction between STIMULUS and TIME (Pz: F_6,138_ = 3.39, p < .01, Oz: F_6,138_ = 3.97, p < .01). At position Oz also a main effect for TIME was evident (F_3,69_ = 3.08, p < .05). Post-hoc results indicated that delta PLI for the target was, at least at Oz, highest in the later three time windows as compared to the first time window (T_23_ > 2.68, p < .05). For the other names, the time course was reversed. Here, delta PLI was higher in the earlier two time windows (Pz: 0-200ms, 200-400ms, Oz: only 200-400ms) as compared to the later ones (400-600ms and 600-800ms; Pz: T_23_ > 2.35, p < .05; Oz: T_23_ > 3.37, p < .01). On both Pz and Oz, the target always presented with a higher phase-locking as compared to the other names (Pz: T_23_ > 2.47, p < .05; Oz: T_23_ > 3.64, p < .05). On Pz, phase locking was also higher for the target as compared to the own name in the last time window (T_23_ = 2.52, p < .05) and on Oz even in the last two time windows (T_23_ > 3.15, p < .05).

*Theta*

Theta PLI behaved very similarly to the passive condition. All midline electrodes showed a main effects for STIMULUS (e.g. Cz: F_2,46_ = 5.06, p < .05; Oz: F_2,46_ = 8.88, p < .01) and (at least by tendency) TIME (e.g. Cz: F_3,69_ = 66.75, p < .001; Oz: F_3,69_ = 2.56, p = .062). On Cz, the earlier two time windows presented with higher PLI values as compared to the last two, and the third time window from 400-600ms still showed higher PLI values than the last time window over all stimuli (T_23_ > 2.56, p < .05). On Oz, post hoc tests did not reveal significant differences between the time windows. Concerning the main effect for STIMULUS, both the own name and the target unfamiliar name showed higher values as compared to the other names (Cz: T_23_ > 2.79, p < .05, Oz: T_23_ > 3.71, p < .01) the own name and the target did, however, not differ.

*Lower Alpha*

Lower alpha PLI was by tendency stronger above anterior compared to posterior sites. ANOVAs revealed main effects for STIMULUS (F_2,46_ > 5.98, p < .05) and TIME (F_3,69_ > 5.23, p < .05) as well as an interaction between STIMULUS and TIME (F6_,138_ > 2.65, p < .05) at all midline electrodes. Concerning the main effect for STIMULUS again a picture similar to the theta band appeared. Post hoc tests for electrode Fz showed that both the own name and the target elicited a stronger phase locking than the other names (T_23_ > 3.00, p < .05). Concerning the time course lower alpha PLI at Fz was generally higher in the first two as compared to the second two time windows (T_23_ > 5.08, p < .05). Concerning the interaction between TIME and STIMULUS, post hoc results for Fz revealed that in the first time window both the own name and the target elicited higher phase locking as compared to the other names. In the second time window from 200-400ms PLI was higher for the target as compared to both the own name and the other names. In the third and fourth time window phase locking in response to the target was still higher when compared to the other names, but not when compared to the own name (all T_23_ > 2.44, p < .05).

3.1.5 PLV: passive condition

*Delta*

In the first three time windows, i.e. from 0 to 600ms, the own name always elicited a more densely connected network as represented by more connections with a significantly higher PLV compared to the baseline interval (McNemar exact p < .05, corrected for multiple comparisons). Only in the last time window from 600-800ms post-stimulus, the number of connections did not anymore differ between the own name and the later target.

Theta and lower alpha PLV did not yield stimulus-specific differences in network density.

3.1.6 PLV: active condition

In the later three time windows from 200-800ms the target name always elicited a more densely connected network as compared to both the own name and the other names. In the first time window from 0-200ms, connectivity was still stronger for the target as compared to the other names but not when compared to the own name. In the later two time windows, i.e. from 400-800ms, presentation of the own name still activated a stronger network as compared to the other names (McNemar exact p < .05). As in the passive condition, theta and lower alpha PLV did not yield stimulus-specific differences in network density.

3.2 Group differences

For reasons of conciseness, only results which indicate group differences will be reported.

2.2.1 ERS/ERD: passive condition

*Delta*

Since delta ERS was topographically a bit more widespread as compared to controls (at least in MCS patients), we decided to also mention Cz results and not only focus on Pz and Oz. Besides a main effect for TIME (Cz: F_3,108_ = 19.36, p < .001; Pz: F_3,108_ = 19.63, p < .001, Oz: F_3,108_ = 5.69, p < .01), electrodes Cz, Pz and Oz interestingly also showed main effects for GROUP (Cz: F_2,36_ = 4.11, p < .05; Pz: F_2,36_ = 5.37, p < .01; Pz: F_2,36_ = 4.61, p < .01).

Post-hoc tests revealed no significant differences between controls and MCS patients on any of the electrodes. UWS patients, on the other hand, exhibited significantly weaker delta ERS than controls at electrodes Cz, Pz and Oz (T_30_ > 2.91, p < .01). Even when compared to MCS, UWS patients showed weaker delta ERS on position Cz (T_13_ = 2.17, p < .05) and by tendency also on Pz (T_6.86_ = 2.20, p = .064). However, no significant interactions with the factor GROUP were obtained.

*Theta*

The ANOVA GROUP (control vs. MCS vs. UWS) x STIMULUS (own name vs “target” vs. other names) x TIME (0-200ms vs. 200-400 ms vs. 400-600 ms vs. 600-800 ms) at position Fz and Cz indicated a main effect of TIME (Fz: F_3,108_ = 20.03, p < .001; Cz: F_3,108_ = 19.55, p < .001). Interestingly, no main effect for GROUP could be observed. This most likely resulted from the similarity between controls and MCS patients concerning the average ERS to any stimulus. To test this assumption, we calculated post-hoc T-tests comparing the overall theta ERS between groups. Indeed, results indicated a tendency toward higher ERS in controls and MCS patients as compared to UWS patients (Fz: T_30_ = 2.01, p = .06 and T_13_ = 1.77, p = .10; Cz: T_30_ = 1.94, p = .06 and T_13_ = 2.08, p = .06), but no difference between controls and MCS patients.

*Lower Alpha*

The ANOVA GROUP (control vs. MCS vs. UWS) x STIMULUS (own name vs. “target” vs. other names) x TIME (0-200ms vs. 200-400 ms vs. 400-600 ms vs. 600-800 ms) at electrode Pz revealed a main effect for TIME (F_3,108_ = 3.42, p < .05) as well as an interaction STIMULUS x GROUP (F_4,72_ = 3.27, p < .05). Post-hoc test showed, that again only controls showed a differential response to the own name, i.e. presented with significantly stronger lower alpha ERD for the own name as compared to the later target and the other names (T_23_ > 2.68, p < .05). The analysis at position Oz did not yield significant results.

3.2.2 ERS/ERD: active condition

*Delta*

Besides a main effect of TIME, the ANOVA GROUP (control vs. MCS vs. UWS) x STIMULUS (own name vs “target” vs. other names) x TIME (0-200ms vs. 200-400 ms vs. 400-600 ms vs. 600-800 ms) for position Cz also revealed main effects for GROUP (F_2,36_ = 5.62, p < .01) as well as interactions between GROUP x TIME (F_6,108_ = 2.20, p < .05) and GROUP x STIMULUS (F_6,72_ = 2.53, p < .05). Analyses at Pz and Oz revealed a similar picture (Pz: main effect for GROUP, F_2,36_ = 10.05, p < .001; interaction GROUP x STIMULUS, F_4,72_ = 4.21, p < .01; interaction GROUP x TIME, F_6,108_ = 2.43, p < .05; Oz: main effect for GROUP, F_2,36_ = 16.89, p < .001; interaction GROUP x STIMULUS, F_4,72_ = 3.59, p < .05; interaction GROUP x TIME, F_6,108_ = 4.51, p < .001)

Concerning post-hoc tests for the main effect for GROUP, overall delta ERS was different between controls and MCS at Oz (T_29_ = 2.89, p < .05) and by tendency also at Fz (T_29_ = 1.88, p = .071). Controls showed higher delta ERS than UWS patients at all electrodes (T_30_ > 2.69 , p < .05). At parieto-occipital electrodes, even MCS patients showed, by tendency, higher delta ERS than UWS patients (Pz: T_7.13_ = 1.91, p = .098; Oz: T_13_ = 1.871, p = .084). Post-hoc results for the interactions with GROUP did not reveal any differences within the group of MCS or UWS patients.

*Theta*

The ANOVA GROUP (control vs. MCS vs. UWS) x STIMULUS (own name vs “target” vs. other names) x TIME (0-200ms vs. 200-400 ms vs. 400-600 ms vs. 600-800 ms) at position Cz again indicated a main effect for TIME (F_3,108_ = 19.55, p < .001). In contrast to the passive condition, here, a trend towards a main effect for GROUP was evident (F_2,36_ = 3.06, p = .06). Post-hoc comparison revealed that controls showed overall higher theta ERS than both MCS (tendency at T_28.17_ = 1.95, p = .06) and UWS (T_28.13_ = 3.17, p < .01) patients. The two patient groups did, however, not differ (T_13_ = 1.67, p = .11).

*Lower Alpha*

The ANOVA GROUP (control vs. MCS vs. UWS) x STIMULUS (own name vs “target” vs. other names) x TIME (0-200ms vs. 200-400 ms vs. 400-600 ms vs. 600-800 ms) for position Oz yielded significant interactions between GROUP and STIMULUS (F_4,72_ = 3.86, p < .01), GROUP and TIME (F_6,108_ = 3.39, p < .01) as well as the three-factorial interaction (F_2,216_ = 3.05, p < .01). Post-hoc tests indicated that in the last time window from 600-800ms lower alpha ERD was strongest in controls compared to MCS (T_29_ = -3.08 p < .05) and UWS (T_29_ = -3.93 p < .05) patients.

Furthermore, in controls ERD for the target was always higher in the later time windows as compared to the earlier time windows (0-200ms < 400-600ms/600-800ms and 200-400ms < 400-600ms/600-800ms and 400-600ms < 600-800ms, T_23_ > 3.409, p < .05). In one post hoc comparison, MCS showed a stronger ERD from 200-400ms as compared to 0-200ms, however, only for other names (T_6_ = 6.83, p < .05)

3.2.3 PLI: passive condition

*Delta*

The ANOVA GROUP (control vs. MCS vs. UWS) x STIMULUS (own name vs “target” vs. other names) x TIME (0-200ms vs. 200-400 ms vs. 400-600 ms vs. 600-800 ms) for position Pz indicated a main effect for STIMULUS (F_2,72_ = 3.61, p < .05), a trend toward a main effect for TIME (F_3,108_ = 2.15, p = .10), a main effect of GROUP (F_2,36_ = 5.44, p < .01) as well as an interaction between GROUP and TIME (F_6,108_ = 4.86, p < .001). Post hoc tests revealed that controls showed higher PLI values as compared to UWS (T_30_ = 4.75, p < .01) but not as compared to MCS patients. However, MCS patients did not differ from UWS patients. Concerning the interaction between TIME and GROUP, in the first time window from 0-200ms general delta ERS was higher in controls than in MCS patients (T_29_ = 2.93, p < .05). As compared to UWS patients, controls showed higher delta ERS in even the first three time windows between 0-600ms (T_30_ > 2.84, p < .05). However, the two patient groups did not differ in any time window.

The same analysis for position Oz showed a similar picture. The ANOVA indicated a main effect for TIME (F_3,108_ = 6.22, p < .01), a trend toward a main effect of GROUP (F_2,36_ = 2.85, p = .071) as well as an interaction between GROUP and TIME (F_6,108_ = 2.47, p < .05). Post hoc test revealed that controls showed higher PLI values than UWS (T_30_ = 3.09, p < .01) but not than MCS patients. MCS patients did, again, not differ from UWS patients. Concerning the interaction between TIME and GROUP, controls showed higher PLI values than UWS patients in the first three time windows from 0-600ms (T_30_ > 2.58, p < .05). Here, controls did not differ from MCS patient and again, the two patient groups did not differ either.

*Theta*

Theta PLI was strongest at Cz, but since controls showed the only stimulus-specific differences for theta PLI at position Oz, we also included Oz in the comparison between groups. The ANOVA GROUP (control vs. MCS vs. UWS) x STIMULUS (own name vs “target” vs. other names) x TIME (0-200ms vs. 200-400 ms vs. 400-600 ms vs. 600-800 ms) yielded a main effect of GROUP (Cz: F_2,36_ = 10.01, p < .001; Oz: F_2,36_ = 4.27, p < .05) as well as an interaction between GROUP and TIME 2.47 (Cz: F_6,108_ = 11.28, p < .001; Oz: F_6,108_ = 3.41, p < .01). At electrode Cz, controls showed a trend towards higher theta PLI compared to MCS (T_29_ = 2.04, p = .051). At closer inspection this difference, however, was only based on the first time window from 0-200ms (T_29_ > 3.39, p < .001). Overall, theta PLI was also higher in controls than in UWS patients (T_30_ = 4.96, p < .001). This effect was based on the first two and the last time windows (T_30_ > 2.16, p < .05). Again, MCS and UWS patients did not differ.

At electrode Oz, post hoc tests revealed that controls showed higher PLI values than UWS (T_30_ = 3.67, p < .01) but not when compared to MCS patients. MCS patients did, again, not differ from UWS patients. Concerning the interaction between TIME and GROUP, controls showed higher PLI values than UWS patients in the first three time windows, i.e. from 0-600ms (T_30_ > 2.26, p < .05). Here, controls did not differ from MCS patients and the two patient groups did not differ either.

*Lower alpha*

The ANOVA GROUP (control vs. MCS vs. UWS) x STIMULUS (own name vs “target” vs. other names) x TIME (0-200ms vs. 200-400 ms vs. 400-600 ms vs. 600-800 ms) for position Fz revealed a main effect of GROUP (F_2,36_ = 13.63, p < .001) as well as an interaction between GROUP and TIME 2.47 (F_6,108_ = 9.93, p < .001). Post hoc test revealed that controls showed higher PLI values as compared to both UWS (T_30_ = 4.67, p < .01) and MCS patients (T_29_ = 3.75, p < .01). Furthermore, MCS patients did not differ from UWS patients. Concerning the interaction between TIME and GROUP, controls showed higher PLI values than both MCS and UWS patients in the first two as well as the fourth time windows (MCS: T_29_ > 2.65, p < .05; UWS: T_30_ > 2.64, p < .05). Again, the two patient groups did not differ.

3.2.4 PLI: active condition

*Delta*

The ANOVA GROUP (control vs. MCS vs. UWS) x STIMULUS (own name vs “target” vs. other names) x TIME (0-200ms vs. 200-400 ms vs. 400-600 ms vs. 600-800 ms) at position Pz revealed a main effect for GROUP (F_2,36_ = 6.56, p < .01) and an interaction between GROUP and STIMULUS (F_4,72_ = 3.47, p < .05). Post hoc test revealed that controls showed higher PLI values than UWS (T_30_ = 4.97, p < .001) but not than MCS patients. However, MCS patients did not differ from UWS patients. Concerning the interaction between STIMULUS and GROUP, controls showed higher PLI values than UWS patients for all three stimulus types (T_30_ > 2.67, p < .05). Controls and MCS patients as well as the two patient groups did not differ.

The same analysis for position Oz revealed a main effect for GROUP (F_2,36_ = 6.96, p < .01) as well as an interaction between GROUP and STIMULUS (F_4,72_ = 5.44, p < .001). Post hoc tests showed that controls presented with higher PLI values than UWS patients (T_30_ = 5.00, p < .001), but were not different from MCS patients. Furthermore, MCS patients did not differ from UWS patients. Concerning the interaction between STIMULUS and GROUP, controls showed higher PLI values than UWS patients for all three stimulus types (T_30_ > 2.67, p < .05) and as compared to MCS patients only for the target name (T_29_ > 2.60, p < .05). The two patient groups did not differ.

*Theta*

The ANOVA GROUP (control vs. MCS vs. UWS) x STIMULUS (own name vs “target” vs. other names) x TIME (0-200ms vs. 200-400 ms vs. 400-600 ms vs. 600-800 ms) at position Cz revealed a main effect of GROUP (F_2,36_ = 8.32, p < .01) as well as an interaction between GROUP and TIME 2.47 (F_6,108_ = 11.17, p < .01). Post hoc test indicated that controls showed higher PLI values as compared to UWS (T_30_ = 4.65, p < .001) but not as compared to MCS patients. The patient groups did not differ. Concerning the interaction between TIME and GROUP, controls showed higher PLI values than MCS patients in the first time window (T_29_ = 3.10, p < .05) and higher values than UWS patients in the first two time windows (T_30_ > 5.39, p < .01). The patient groups, however, did not differ.

Again, the same analysis was conducted for position Oz. The ANOVA revealed a main effect of GROUP (F_2,36_ = 6.78, p < .01) as well as an interaction between GROUP and TIME (F_6,108_ = 2.62, p < .05). Post hoc test revealed that controls showed higher PLI values than UWS (T_30_ = 4.45, p < .001) but no difference to MCS patients. Interestingly, MCS patients also showed by tendency higher theta PLI as compared to UWS patients (T_13_ = 2.07, p = .06). Concerning the interaction between TIME and GROUP, controls showed higher PLI values than UWS patients in all four time windows (T_30_ > 2.39, p < .05). Again, controls did not differ from MCS patients. Also MCS patients showed higher PLI values than UWS patients in the second time window from 200-400ms (T_13_ = 2.69, p < .05).

*Lower alpha*

The ANOVA GROUP (control vs. MCS vs. UWS) x STIMULUS (own name vs “target” vs. other names) x TIME (0-200ms vs. 200-400 ms vs. 400-600 ms vs. 600-800 ms) for position Fz revealed a main effect of GROUP (F_2,36_ = 12.39, p < .001) as well as an interaction between GROUP and TIME (F_6,108_ = 4.50, p < .001). Post hoc test revealed that controls showed higher PLI values as compared to both UWS (T_30_ = 4.75, p < .001) and MCS (T_29_ = 3.26, p < .01) patients. Concerning the interaction between TIME and GROUP, controls showed higher PLI values than UWS patients in all four time windows (T_30_ > 2.38, p < .05). In comparison to MCS patients, controls only showed higher values in the first and the last time window (T_29_ > 2.96, p < .05).

3.2.5 PLV: passive condition

Chi^2^ tests revealed a higher network density in controls as compared to both MCS and UWS patients in response to the own name (HC vs. MCS: the number of significant connections was higher in the three time windows from 0-600ms; HC vs. UWS: the number of connections was higher in all four time windows (χ^2^ > 13.31, p < .05). On only a few occasions, network density was also higher in controls as compared to both patient groups in response to the other stimuli (χ^2^ > 6.41, p < .05).

Between patient groups, only on one occasion network density was higher for MCS than for UWS patients (other names, 200-400ms, χ^2^ = 6.55, p < .05).

Within patient groups McNemar tests did not reveal significant differences between stimuli in any of the four time windows.

Since theta and lower alpha did not reveal stimulus-specific differences in network connectivity in controls, theta and lower alpha connections were not subjected to a group analysis.

3.2.6 PLV: active condition

Chi^2^ tests again revealed a higher network density in controls as compared to both MCS and UWS patients in response to the target name in all four time windows (all χ^2^ > 13.70, p < .05). Furthermore, the number of connections was also higher in controls as compared to patients regarding the response to the own name (as compared to MCS in the three time windows from 200-800ms and as compared to UWS in all four time windows (all χ^2^ > 6.56, p < .05). On a few occasions, network density was also higher in controls as compared to both patient groups in response to the other names (all χ^2^ > 6.38, p < .05).

Between patient groups, again, on one occasion network density for the target name was higher for MCS as compared to UWS patients (400-600ms, χ^2^ = 9.42, p < .05).

Within patient groups McNemar tests did not reveal significant differences between stimuli in any of the four time windows.

Again, since theta and lower alpha did not reveal stimulus-specific differences in network connectivity in controls, theta and lower alpha connections were not subjected to a group analyses.

**Supplementary figure captions**

**Supplementary figure 1. Delta, theta and lower alpha PLI in response to the different stimuli in the passive and the active condition in healthy controls and patient groups.** While in controls delta and theta PLI indicated the focus of attention in the passive condition, delta and lower alpha PLI were pronounced for the target in the active condition. Additionally, general theta PLI in the active condition was by tendency higher in MCS as compared to UWS patients. Red rectangles indicate the time windows in which the own name showed significantly stronger PLI as compared to all other names, or the target showed higher PLI as compared to both the own and the other names. *p < .05 or **^t^**p < .10

**Supplementary figure 2. ERS/ERD scalp maps for MCS and UWS patients for the delta, theta and lower alpha band for the active condition**. Delta ERS at parieto-occipital sites independent of the stimulus type was by tendency higher in MCS as compared to UWS patients. Brackets indicate stronger ERS in MCS as compared to UWS patients at ^t^p < .10.
