# Supplementary material for: Event-related EEG power modulations and phase connectivity indicate the focus of attention in an auditory own name paradigm
Source: J Neurol. 2016 May 23;263:1530–43. doi: 10.1007/s00415-016-8150-z (PMC4971049; doi:10.1007/s00415-016-8150-z)
Supplement: Supplementary file 6 — Supplementary material 6 (DOCX 15 kb) [file 415_2016_8150_MOESM6_ESM.docx]

Table 1

| Patient ID | Age(years) | Sex | Etiology | Time since injury | | Clinical assessment | CRS-R total score | CRS-R auditory score |
| --- | --- | --- | --- | --- | --- | --- | --- | --- |
| MCS1 | 57 | m | Anoxic brain lesion after myocard infarction | 11 years 3 months | | MCS | 12 | 3 |
| MCS2 | 45 | m | Subdural hematoma, subarachnoidal hemorrhage,skull fracture | 1 year |  | MCS | 8 | 0 |
| MCS3 | 56 | w | Hypoxia | 7 years 1 month | | MCS | 12 | 3 |
| MCS4 | 73 | m | Intracerebral hemorrhage | 8 months |  | MCS | 17 | 3 |
| MCS5 | 21 | m | Anoxic brain lesion after mixed intoxication | 2 years 4 months | | MCS | 13 | 3 |
| MCS6 | 50 | w | Subdural hematoma after violent crime | 9 years 5 months | | MCS | 14 | 4 |
| MCS7 | 30 | m | Trauma | 9 years 3 months | | MCS | 13 | 3 |
| UWS1 | 20 | m | SSPE (syn. Bogaert encephalitis) | 3 years |  | UWS | 3 | 1 |
| UWS2 | 51 | w | Subdural hematoma, ruptured aneurism, hydrocephalus | 4 years 1 month | | UWS | 4 | 0 |
| UWS3 | 48 | m | Hypoxia | 9 years 3 months | | UWS | 5 | 0 |
| UWS4 | 52 | m | Subdural hematoma, osteoclastic trepanation | 12 years 3 months | | UWS | 8 | 2 |
| UWS5 | 53 | m | Trauma | 1 year 1 month | | UWS | 4 | 0 |
| UWS6 | 58 | w | Ruptured aneurism | 2 years 4 months | | UWS | 4 | 0 |
| UWS7 | 62 | m | Hypoxia after cardiopulmonary resuscitation | 2 years 8 months | | UWS | 4 | 2 |
| UWS8 | 41 | w | Deceleration trauma with cortical and subcortical contusions | 12 years 8 months | | UWS | 7 | 2 |
|  |  |  |  |  | |  |  |  |

Demographic data of patients with Minimally Conscious State (MCS) and Unresponsive Wakefulness Syndrome/Vegetative State (UWS/VS).
